# Prenatal screening of Down syndrome in assisted reproductive techniques pregnancies: A systematic review

**DOI:** 10.18502/ijrm.v23i3.18773

**Published:** 2025-06-10

**Authors:** Fatemeh Zahra Meamar, Mitra Savabi-Esfahani, Tahmineh Farajkhoda

**Affiliations:** ^1^Department of Midwifery and Reproductive Health, Faculty of Nursing and Midwifery, Reproductive Sciences and Sexual Health Research Center, Isfahan University of Medical Sciences, Isfahan, Iran.; ^2^Department of Midwifery and Reproductive Health, Faculty of Nursing and Midwifery, Isfahan University of Medical Sciences, Isfahan, Iran.; ^3^Research Center for Nursing and Midwifery Care, Non-Communicable Diseases Research Institute, Department of Midwifery, School of Nursing and Midwifery, Shahid Sadoughi University of Medical Sciences, Yazd, Iran.

**Keywords:** Down syndrome, Prenatal diagnosis, Maternal serum screening tests, Assisted reproductive technique.

## Abstract

**Background:**

The interpretation of Down syndrome screening results in assisted reproductive technology (ART) pregnancies is challenging. Despite the high psychological burden that false positive results impose on parents, studies that have addressed interpretation of both serum and sonographic markers in both rounds of screening for Down syndrome diagnosis in post-ART pregnancies are limited.

**Objective:**

This review study investigated the types of serum screening and imaging for prenatal diagnosis of Down syndrome in ART pregnancies to know and correctly interpret the results of prenatal screenings in these pregnancies.

**Materials and Methods:**

In this systematic review, an extensive search was conducted in Persian and English in PubMed, Web of Science, Scopus, SID, and Google Scholar without any time limit until January 2024 using appropriate keywords. PRISMA guideline, STROBE, and CONSORT checklists were used.

**Results:**

Review of 30 articles showed in the first screening, pregnancy-associated plasma protein-A was significantly lower than normal values compared to spontaneous pregnancies, while free beta-human chorionic gonadotropin, especially in the in vitro fertilization (IVF) and intracytoplasmic sperm injection (ICSI) was significantly higher. Some studies also indicated an increase in nuchal translucency in the first trimester of pregnancies resulting from ART. Biochemical markers of second screening, in some studies, showed an increase in inhibin-A, a decrease in α-fetoprotein, and unconjugated estriol were evident compared to normal values.

**Conclusion:**

Marker levels may be different for the presence of ovulation-stimulating hormones, multiple corpora lutea, twins or multiplets, type of IVF, and changes in egg cytoplasm in ICSI. Study suggests concentration of maternal serum markers, especially free beta-human chorionic gonadotropin and pregnancy-associated plasma protein-A, should be adjusted differently for each ART (IVF and ICSI separately).

## 1. Introduction

Down syndrome (trisomy 21) is the most prevalent chromosomal abnormality in neonates with a higher chance of survival than other chromosomal disorders. Its overall prevalence is about 10 cases per 10,000 live births around the globe (1). Due to the high costs of caring for these children and the high burden of medical, emotional, and psychological services for the family, the life quality of these people's families is also influenced (2). Thus, prenatal diagnosis of these diseases and termination of pregnancy at the right time can reduce the amount of suffering caused by their occurrence.

Among the various types of prenatal screening protocols, the most widely used during the first trimester of pregnancy is the combination of nuchal translucency (NT) measurement and serum level measurement of free beta-human chorionic gonadotropin (fβHCG) and pregnancy-associated plasma protein-A (PAPP-A), the so-called integrated screening. The most widely used second trimester screening test is the quad test, which uses the mother's age, serum beta-human chorionic gonadotropin (βHCG), alpha-fetoprotein (AFP), unconjugated estriol (uE3), and inhibin A. In the combined screening protocol, the results of the first and second trimesters are integrated, and the sensitivity of Down syndrome diagnosis reaches 94–96%. Yet, it is still associated with 5% false-positive results (3). Assisted reproductive technology (ART) pregnancies may have levels of biochemical markers that are different, especially during the second trimester, compared to spontaneous pregnancies (4). The false-positive rate for diagnosing Down syndrome after ART pregnancies is also 5% in both screening rounds (5), leading to unnecessary invasive diagnostic measures in some cases (6).

Pregnancy can be a challenging and stressful period for women who have conceived through ART (7). One of the concerns of these couples is to perform prenatal screenings because there is a possibility that the screening in these women may be wrong. In other words, it may have been a false positive, and as a result, it will cause them a lot of anxiety. Moreover, invasive diagnostic procedures such as amniocentesis and chorionic villus sampling are necessary for them (8–10). In contrast, having screening methods to detect chromosomal disorders before birth is one of the reproductive health rights of couples, and they have the right to have a healthy child (11). Interpreting the results of chromosomal disease screenings using serum and sonographic markers after ART pregnancies is challenging. Firstly, the mean age of mothers is higher than spontaneous pregnancies. Secondly, if it leads to twins or multiples, relying on the mother's serum markers may show abnormal results despite the health of the fetus (12).

Considering that the majority of chromosomal problems in pregnancies resulting from ART have not been proven so far, but the serum markers of Down syndrome have been reported in some studies to be different in pregnancies resulting from ART compared to natural pregnancies, and considering that nowadays ART includes a large group of methods that have many differences, also knowing that couples with infertility have succeeded in experiencing pregnancy after paying a lot of financial and psychological costs and have the right to health diagnostic methods before birth, and having a normal fetus and baby, this study was designed with the aim of systematic review of types of prenatal imaging and serological screenings for the diagnosis of Down's syndrome in pregnancies resulting from assisted reproductive technologies using available valid documents in order to know and correctly interpret the results of prenatal screenings in these pregnancies.

## 2. Materials and Methods 

The present study is a systematic review. To determine the time interval and keywords, first, an extensive search was conducted in Persian and English in PubMed, Web of Science, Scopus, SID, and Google Scholar databases. Accordingly, to obtain a comprehensive number of documents, a search was made without applying a time limit until January 2024 (Table I). To strengthen the obtained information, the characteristics of the papers were also examined (Table II) (13). Further, to access all the information available on the subject, no limitations were considered in terms of the methodology of the papers, and every document in Persian and English that focused on prenatal screening for Down syndrome in post-ART pregnancies was considered. The papers in languages other than Persian and English were not included in the study. In addition, papers whose full text were not available or studies that focused on other trisomies, such as trisomy 13 and 18 were also excluded from the study. Based on the inclusion criteria, searching documents and evaluating the titles of the papers were conducted by a researcher. Then, the abstracts and titles of the resulting papers were evaluated based on the inclusion criteria by 2 other members of the research team. If 2 researchers had different opinions in evaluating the abstracts of papers, a third researcher decided on the inclusion of that paper. Subsequently, to check the quality of papers after preparing their full text, the STROBE checklist was used to evaluate descriptive studies. Besides, `15.5' was considered as the minimum obtainable score for the STROBE checklist (14). The CONSORT scale quality scoring system was used for clinical trials. The total score of the scale based on these statements is 5. Papers with a score of 3 or higher were included in the study (15).

### Search strategy

A systematic review was conducted in Persian and English in PubMed, Scopus, SID, Google Scholar, and Web of Science databases. The search was performed in PubMed using MeSh terms in combination with keywords (Downs Syndrome) or (Mongolism) or (47,XY,+21) or (47,XX,+21) or (Trisomy 21) and (screening) or (Maternal) (Serum Screening Tests) or (Prenatal Diagnosis) and (Assisted Reproductive Technique) or (Fertilization in Vitro) or (Sperm Injections, Intracytoplasmic) or (Ovulation Induction) or (Superovulation) or (Embryo Transfer) or (Donor Conception). This search strategy was adapted to other databases. For complete searching, all citations were imported into the EndNote basic database (Table I). Based on a comprehensive list of possible synonyms for each term, a search was followed up to April 2023. Synonyms combined with the conjunction “OR" and a search for all 4 characteristics together with “AND" yielded a list of sources used to search for related papers. Data extraction of eligible papers was conducted independently by 2 researchers.

**Table 1:** Search strategies and databases

**Database**	**Search strategy**	**Results**
**PubMed**	((((((((((((((((((Downs Syndrome [MeSH Terms]) OR (Mongolism [MeSH Terms]) OR (47,XY,+21 [MeSH Terms]) OR (47,XX,+21 [MeSH Terms] OR (Trisomy 21 [MeSH Terms]) AND(screening [MeSH Terms]) OR (Maternal Serum Screening Tests [MeSH Terms]) OR (Prenatal Diagnosis [MeSH Terms]) AND(Assisted Reproductive Technique [MeSH Terms]) OR (Fertilization in Vitro [MeSH Terms]) OR (Sperm Injections, Intracytoplasmic [MeSH Terms] OR (Ovulation Induction [MeSH Terms] OR (Superovulation [MeSH Terms] OR (Embryo Transfer [MeSH Terms]) OR (Donor Conception [MeSH Terms])	58,321
**Scopus**	TITLE-ABS-KEY (“Downs Syndrome"( OR(Mongolism") OR (“Trisomy 21") OR (“47,XY,+21") OR (“47,XX,+21") AND TITLE-ABS-KEY (“screening") OR (“Maternal Serum Screening Tests") OR (“Maternal Serum Screening Tests") AND TITLE-ABS-KEY (“Assisted Reproductive Technique") OR (“Fertilization in Vitro") OR (“Sperm Injections, Intracytoplasmic") OR (“Ovulation Induction") OR (“Superovulation") OR (“Embryo Transfer") OR (“Donor Conception")	9479
**Web of Science**	TI=(“Downs Syndrome") OR (“Mongolism") OR (“Trisomy 21") OR (“47,XY,+21") OR (“47,XX,+21") AND TI=(“screening") OR (“Maternal Serum Screening Tests") OR (“Maternal Serum Screening Tests") AND TI=(“Assisted Reproductive Technique") OR (“Fertilization in Vitro") OR (“Sperm Injections, Intracytoplasmic") OR (“Ovulation Induction") OR (“Superovulation") OR (“Embryo Transfer") OR (“Donor Conception")	3450

### Ethical Considerations

This systematic review has been approved by the Ethics Committee of Yazd Reproductive Sciences Institute, Shahid Sadoughi University of Medical Sciences, Yazd, Iran (Code: IR.SSU.RSI.REC.1403.001, project code: 17649).

## 3. Results

**Figure 1:**
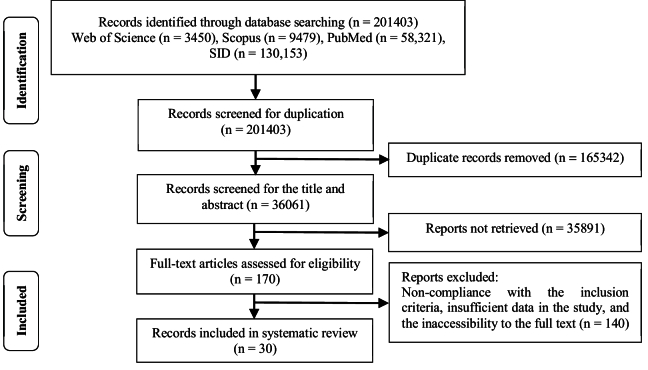
PRISMA flowchart for selecting papers.

Out of 201,403 eligible articles identified, 165,342 were duplicates and were not included in the study. 2 independent reviewers evaluated the titles and abstracts of 36,061 studies. Moreover, 35,891 papers had unrelated reports and were excluded. In total, 170 eligible papers were analyzed by both reviewers. Although some studies met the inclusion criteria, they were excluded because the complete article was not available. Of these papers, 140 were excluded due to insufficient data and lack of access to complete data. Thus, 30 papers were finally included in the study. The search and selection process are presented in figure 1 using the preferred reporting items for systematic reviews and meta-analyses flowchart.

### First-time screenings for Down syndrome in post-ART pregnancies

In the first screening test for Down syndrome, performed in the first trimester of gestation between 11 ${}^{\text{th}}$ and 13 ${}^{\text{th}}$ wk and 6 days, 2 types of measurements were used along with the mother's age to calculate the risk of Down syndrome (trisomy 21), trisomy 13 and 18. One of these measurements was through ultrasound and NT measurement, and the other was through the measurement of dual blood biomarkers, namely PAPP-A and fβhCG, while also considering the mother's age. In the past, there was another marker in the screening of the first trimester of Down syndrome, that is, the visibility of the nasal septum (nasal bone) in sonography, which is now excluded from the first screening protocol. In relation to the difference in biochemical markers between spontaneous pregnancies and post-ART pregnancies, we found that 16 studies showed statistically significant differences in reducing the PAPP-A index (abnormal values < 0.1 MOM) (4, 6, 16–29), 8 studies showed increased fβhCG index (abnormal values > 0.1 MOM) in most ART methods, especially in vitro fertilization (IVF) and intracytoplasmic sperm injection (ICSI) (4, 17, 18, 22, 25, 27, 28, 30) and 1 study showed an increase in the level of ADAM12-s (a disintegrin and metalloprotease) (abnormal values > 0.1 MOM) (29) in the first trimester screening; nonetheless, 5 studies rendered false-positive results as probable in the biochemical markers of the first trimester (4, 16, 21, 22, 31). An increased NT thickness (abnormal values of NT above 3 mm) was observed in 4 studies following some ART methods (6, 24, 30, 31). However, one study did not observe any difference in NT (25). These serum and ultrasound NT indicators revealed a positive screening in terms of Down syndrome. One study has recommended performing the cff DNA serum screening method (32) to reduce false-positive reports and thus prevent additional invasive tests, and one study has suggested non-invasive prenatal testing (NIPT), especially in twins (33).

### Second round screenings for Down syndrome in post-ART pregnancies

The second screening test for Down syndrome was performed at the gestational age of 14 wk and 1 day to 20 wk by measuring 4 blood biomarkers (quad markers) including βhCG, uE3, inhibin A, and AFP. Regarding the difference in biochemical markers between spontaneous pregnancies and post-ART pregnancies, 4 studies reported an increase in βhCG (abnormal values > 0.1 MOM) (6, 21, 34, 35), 2 studies indicated a decrease in uE3 (abnormal values < 0.1 MOM) (21, 34), 2 studies reported a decrease in AFP (abnormal values < 0.1 MOM) (6, 19), and 2 studies indicated increased inhibin A (abnormal values > 0.1 MOM) (21, 26) in the second round of screening in post-ART pregnancies. Nevertheless, 3 studies considered false-positive results of the second screening biomarkers of Down syndrome as possible (21, 34, 35). Following these false positive results, 1 study performed a consecutive integrated test (36) and 10 studies suggested the correction of defined values for Down syndrome serum markers in ART pregnancies (4, 18, 21, 24, 25, 27, 29, 35–38). Yet, 5 studies did not consider the reported results to be false positives and did not see the need to adjust in serum markers; they did not report any difference in diagnostic screenings between spontaneous pregnancies and post-ART pregnancies (17, 34, 39–41). Even Hui stated in their study that couples undergoing ART should be counseled regarding the increased risk of adverse pregnancy outcomes and prenatal outcomes (6).

**Table 2:** Characteristics of studies

**Author, year (Ref)**	**Place of study**	**Type of study**	**Aim of study**	**Sample volume**	**Results**
**Amor ** ***et al.*** **, 2009 (16)**	Australia	Cohort	Analysis of first trimester screening results of singleton pregnancies in ART and non-ART pregnancies.	1739 ART pregnancies and 50,253 normal pregnancies.	In general, PAPP-A levels were found to be significantly lower in ART pregnancies than in the control group (p < 0.001). ART pregnancies have reduced PAPP-A levels, which leads to an increased chance of receiving a false positive result and having to undergo CVS/amniocentesis. Low PAPP-A may indicate impaired early implantation in some forms of ART.
**Anckaert ** ***et al.*** **, 2008 (4)**	Brussels	Retrospective cohort	Comparison of Down syndrome screening markers in the first trimester including fβhCG and PAPP-A in wk 11-14 of pregnancy in normal pregnancy and post-ART pregnancies.	4088 women with normal pregnancy IVF = 59, ICSI = 163, FET = 31	Lower serum PAPP-A levels were found in pregnancies after IVF and ICSI and higher serum fβhCG levels in non-male factor infertility at 11-14 wk of gestation. Serum PAPP-A was lower in ART pregnancies with “female factor infertility” (0.93, p < 0.009) and in ART pregnancies with “male factor infertility” (0.87, p < 0.005). It is important to determine whether fβhCG MoM values should be corrected for non-male factor infertility, as it is best to avoid the high false positive rate in this group.
**Barkai ** ***et al.*** **, 1996 (34)**	Israel	Cohort	Serum levels of AFP, hCG, and uE3 were investigated after ART (second screening).	In 1632 women who had ovulation induction and 327 women who had IVF.	Among those who underwent ovulation induction, hCG levels were significantly increased and uE3 levels were decreased, masking a significant increase with clomiphene treatment (1.05 MOM) and a significant decrease with pergonal (0.93 MOM). A statistically significant decrease in uE3 was observed among women who underwent IVF. Those fertilized with donated eggs had significantly higher levels of AFP and uE3 than when their own eggs were used. It can be assured that the positive Down syndrome screening test in these women is unlikely to be due to their assisted reproduction (not a false positive).
**Bellver ** ***et al.*** **, 2005 (17)**	Spain	Cohort	Comparison of Down syndrome screening markers in the first trimester, including fβhCG and PAPP-A in wk 11-14 of pregnancy in normal pregnancy and post-ART pregnancy.	Normal pregnancy (498 people), ovulation induction (97 people), IVF (47 people), ICSI (222 people), and egg donation (190 people).	PAPP-A levels were only different between OS and IVF pregnancies (p < 0.05). fβhCG values in IVF and ICSI were above those found in normal pregnancies, (p = 0.02). The data collected so far show that biochemical markers of the first trimester either do not need to be adjusted because they have no effect on FPRs in ART-derived pregnancies or the effect is very small. Thus, the first trimester of integrated ultrasound and biochemical screening for Down's syndrome in assisted reproductive pregnancies seems possible. That is, it is not a false positive.
**Bonnin ** ***et al.*** **, 2017 (18)**	France	Retrospective cohort	Investigation of maternal serum markers in donor pregnancies (OD) and ART compared to normal pregnancies.	In 614 OD and 1921 ART pregnancies vs. 7268 normal pregnancies.	Serum βhCG levels were significantly increased in the OD group for both trimesters (first trimester p < 0.001 and second trimester p < 0.001). In the ART group, serum PA PP-A levels were significantly lower (p < 0.001). Therefore, maternal serum markers for DS screening in ART and OD pregnancies changed significantly. Since these markers are also considered for obstetric complications, the rationale for applying correction factors is questionable.
**Costa ** ***et al.*** **, 2018 (32)**	France	Prospective interventional	Performance of cell-free DNA compared to MSS in pregnancy with or without ART.	472 patients with spontaneous pregnancies and 322 post-ART pregnancies.	Overall, the FPR and PPV were 6.6% and 8.8% for MSS but 0% and 100% for cfDNA. The MSS FPR and PPV were significantly poorer in the ART group (11.7% and 2.6%) than in the SP group (3.2% and 21.1%). The study findings suggest that cfDNA is better considered for primary screening, especially in post-ART pregnancies.
**Gjerris ** ***et al.*** **, 2008 (42)**	Denmark	Cohort	Description of the use and outcomes of invasive prenatal diagnostic tests in a Danish national IVF/ICSI cohort.	6122 IVF patients, 2087 ICSI patients, and 322 IVF-ICSI patients.	Chromosomal aberrations or chromosomal abnormalities were more common in the group treated with ICSI compared to the group treated with IVF, even though the mean age of the mother was lower (p < 0.0001). Paternal characteristics may explain this difference and emphasize the importance of genetic evaluation of men before performing ICSI.
**Hsu ** ***et al.*** **, 1999 (19)**	Chang Gung	Cohort	Investigating the effect of IUI on the screening results of maternal serum for Down syndrome.	43 women with IUI pregnancies and 4507 women with spontaneous pregnancies.	Maternal serum AFP was significantly lower in the IUI group than in the control group. No significant difference between serum free βhCG levels in the 2 groups. Also, IUI pregnancy was associated with low AFP levels. Determining the cause of this mechanism requires further study.
**Hui 2014 (6)**	Hong Kong	Longitudinal cohort	Investigation of AFP and hCG in the second trimester of pregnancy in ART pregnancies (second screening).	—	A decrease was observed in AFP levels in maternal serum in pregnancies with fresh fetuses along with an increase in hCG levels in maternal serum and amniotic fluid in pregnancies with frozen fetuses. In the first trimester, PAPP-A significantly decreased in assisted reproductive pregnancies. Apart from biochemical markers, nuchal translucency was also increased in these singleton pregnancies. Couples undergoing ART should be counseled regarding the increased risk of adverse pregnancy outcomes and prenatal outcomes (i.e., no false positives).
**Hui ** ***et al.*** **, 2005 (20)**	Hong Kong	Observational	Investigating the effect of ICSI, freezing and thawing of embryos on PAPP-A and βhCG levels.	149 women who conceived with fresh embryos after assisted reproduction (92: IVF and 57: ICSI), 85 women who conceived with frozen embryos (54: IVF and 31: ICSI), and 401 spontaneously conceived control women.	The mean PAPP-A of mothers in ICSI pregnancies in fresh and frozen-thawed embryo subgroups (0.70 and 0.66 MoM, respectively) and in fresh IVF embryo subgroups (0.83 MoM), significantly decreased compared to the control group (1.00 MoM). Maternal serum βhCG levels were significantly lower in the fresh fetus subgroup (0.83 MoM) compared to the other 3 groups.
**Lai ** ***et al.*** **, 2003 (31)**	Taipei China	Cohort	To assess whether singleton pregnancies with IUI affect first-trimester screening outcomes for Down syndrome compared to normal pregnancies.	49 IUI and 3059 singleton normal pregnancies.	In IUI pregnancies, nuchal translucency thickness and PAPP-A levels were significantly higher. Free βhCG levels were not statistically different between the 2 groups. Singleton pregnancies achieved with IUI have a higher positive screening rate. Not only advanced maternal age, but also exogenous hormones given during the ovulation induction process and after fertilization may be an important factor in positive screening results.
**Lambert-Messerlian ** ***et al.*** **, 2006 (21)**	USA	Multi-centered clinical trial	Investigating Down syndrome screening markers in the first and second trimesters and determining the positive screening rate in pregnancies conceived using ART.	IVF-OI (278) IUI-OI (323) IUI (247) IVF-OI-ED (59) IVF-ED (56) IVF-OI (278) IUI-OI (323) IUI (247) IVF-OI-ED (59) IVF-ED (56)	Serum PAPP-A levels decreased in the IUI-OI group. While serum inhibin A levels increased and estriol decreased in the second trimester in all ART pregnancies, serum hCG was also higher in IVF and IUI pregnancies without ED, and AFP was increased in ED pregnancies. The second trimester screening positivity rate was significantly higher than expected for ART pregnancies. Therefore, ART may be able to alter the levels of second trimester markers and affect the rate of reporting a positive outcome, necessitating different adjustments to second trimester screening depending on the type of ART.
**Lambert-Messerlian ** ***et al.*** **, 2014 (37)**	USA	Clinical trial	Comparison of Down syndrome cfDNA test in women with natural pregnancies with ART pregnancies.	632 euploid (5.2% ARTs) and 73 Down syndrome.	There was no difference for circulating cfDNA in euploid (p = 0.70) or Down syndrome (p = 0.58) pregnancies. Maternal plasma appears to work similarly for Down syndrome in ART and natural pregnancies. Minor modifications to z-scores or their interpretation may be useful in ART-conceived pregnancies.
**Lee ** ***et al.*** **, 2018 (43)**	Australia	Cohort	Are the fetal fraction, test failure rate, and PPV of cffDNA testing different in singleton IVF fertilization compared to spontaneous pregnancy?	4633 (82.4%) spontaneous pregnancies and 992 (17.6%) IVF pregnancies.	The rate of test failure was higher in pregnancies after ART (p < 0.001). The PPV for trisomy 21 in IVF fertilization was high (100.0%), while the test failure rate for other trisomies was lower in IVF pregnancies. In general, positive results require cautious interpretation.
**Liao ** ***et al.*** **, 2001 (22)**	London	Cohort	Studying the impact of assisted reproductive techniques on first trimester screening for Down syndrome markers.	220 cases of IVF and 1233 singleton pregnancies with spontaneous pregnancies.	In IVF pregnancies, higher serum free βhCG levels and lower PAPP-A were observed. However, NT did not change. Therefore, in the FPR report in IVF pregnancies, we may see an increase of 1.2% compared to natural pregnancies.
**Matilainen ** ***et al.*** **, 2011 (23)**	Finland	Retrospective cohort	Investigation of PAPP-A in post-ART pregnancies.	176 cases of ICSI; 282 other ART methods; 24,783 spontaneous pregnancies.	PAPP-A statistically significantly decreased in IVF/ICSI-induced pregnancies. Serum markers should be corrected in post-ART pregnancies to reduce the FPR.
**Maymon and Shulman, 2004 (24)**	Israel	Longitudinal cohort	To evaluate the characteristics of the markers that make up the integrated test and its FPR among a preselected group of pregnancies affected by IVF.	99 healthy singletons from women pregnant with IVF and 1781 individuals with spontaneous pregnancies.	The IVF group had lower PAPP-A values (p < 0.05) and higher NT values (p < 0.05), respectively. According to the integrated test, a higher IVF pregnancy rate was defined as screening positive (6.1% vs. 3.7%), although the values did not reach statistical significance. Serum markers should be corrected in post-ART pregnancies to reduce the FPR.
**Muller ** ***et al.*** **, 2003 (44)**	France	Retrospective cohort	Evaluation of the effect of IVF and ICSI on total hCG, free βhCG, AFP and uE3 as a marker for maternal serum screening for trisomy 21 in the 2 ${}^{\text{nd}}$ trimester.	1515 singleton pregnancies (970: IVF, 545: ICSI) and control group (21014).	No significant difference was reported in maternal serum marker levels between the assisted reproductive technology and control groups. Maternal age also had no effect on the positive screening rate for trisomy 21 screening between the 2 groups (no false positives).
**Oktem ** ***et al.*** **, 2010 (40)**	Turkey	Retrospective case series	Comparison of first-trimester screening for Down syndrome as integrated measurement of NT, free βhCG, and PAPP-A in post-ART pregnancies to spontaneous pregnancies.	972 spontaneous pregnancies without complications and 71 ART pregnancies.	Free βhCG, PAPP-A and NT of ART pregnancies were not significantly different from non-ART pregnancies (p > 0.05). These results suggest that first-trimester screening profiles among ART-uncomplicated singleton pregnancies are similar to those in non-ART pregnancies where gestational age and weeks are comparable (i.e., there are no false positives).
**Pastorino ** ***et al.*** **, 2009 (36)**	Italy	Case series	Performance evaluation of a step-by-step integrated sequential test in ART pregnancies.	72 singleton pregnancies and 16 twin pregnancies.	10% FPR at singletons and 7% in twin pregnancies. Yet, this approach can avoid a significant number of unnecessary amniocentesis procedures.
**Räty ** ***et al.*** **, 2002 (35)**	England	Observational study	This study aimed to compare the level of βhCG and free AFP of the mother in the middle trimester of ART pregnancies and Non-ART pregnancies in trisomy 21 screening (second round).	58 IVF pregnancies, 32 ICSI and 26 singleton pregnancies with FET and a control group of 6548 spontaneous singleton pregnancies.	The overall FPR in Down syndrome screening was 19% in ART pregnancies, which was highest (30.8%) in the FET group. Free βhCG multiplets statistically significantly increased only in the FET group (1.33 MoM; p = 0.012). In the IVF group, there is a positive correlation between the number of embryos transferred and the levels of markers, while there is no significant correlation between the amount of gonadotropin medication and the levels of markers. Therefore, the overall FPR in serum screening for trisomy 21 in ART pregnancies is high, leading to an increase in the use of invasive diagnostic procedures.
**Räty ** ***et al.*** **, 2000 (38)**	Finland	Cohort	Comparison of AFP and βhCG levels between spontaneous and IVF twin pregnancies (second cycle).	From 19-310 pregnancies, 145 twin pregnancies.	βhCG levels were higher in twin pregnancies resulting from IVF than in spontaneous twins (p = 0.08), and this difference should be considered in Down syndrome screening to avoid false positive reporting.
**Rice ** ***et al.*** **, 2005 (45)**	Canada	Cohort	Investigating whether the results of maternal serum triple marker screening in the second trimester are different between IVF pregnancies and natural pregnancies.	88 IVF pregnancies with 596 natural pregnancies.	No statistically significant difference in analyte level or FPR of Down syndrome was observed between IVF and natural pregnancies. IVF patients can be counseled about maternal serum triple marker screening in the same way as naturally conceived pregnancies. It is not a false positive.
**Taheripanah ** ***et al.*** **, 2019 (25)**	Iran	Cohort	Comparison of βhCG and PAPP-A levels between natural and IVF pregnancies during 11-13 wk.	300 pregnancies with IVF and 700 spontaneous pregnancies.	In IVF pregnancies, PAPP-A (p = 0.026) decreased and βhCG (p = 0.030) increased. However, NT and CRL markers did not differ significantly between groups (p > 0.05). This finding can be related to different mating in the ICSI method due to changes in the egg cytoplasm. Thus, these markers may need to be adjusted in ART concepts.
**Tul and Novak-Antolič, 2006 (26)**	Italy	Retrospective cohort	Identifying the trend of changes in serum marker levels of Down syndrome after different methods of assisted reproduction.	1098 women with singleton pregnancy: 130 after IVF-ET, 54 after ICSI, and 914 after spontaneous pregnancy.	After IVF-ET and ICSI, PAPP-A decreased and inhibin A increased in women compared to women after spontaneous fertilization. As the number of retrieved eggs increased, PAPP-A decreased and inhibin-A increased, which may be due to the presence of multiple corpora lutea.
**Yang ** ***et al.*** **, 2022 (33)**	China	Observational study	Investigating the performance and impact of NIPS on twin pregnancies.	747 twin pregnancies.	The incidence of trisomy 21 among twin pregnancies that underwent ART was 0.84%. The performance of NIPS among twin pregnancies after ART in this study is very accurate, especially the birth of twins with abnormalities can be prevented.
**Bender ** ***et al.*** **, 2010 (27)**	Germany	Retrospective cohort	Comparison of first-trimester serum markers in IVF and ICSI pregnancy with spontaneous pregnancy.	110 IVF pregnancies and 331 ICSI pregnancies with 1431 spontaneous pregnancies.	PAPP-A serum levels were lower in IVF and ICSI pregnancies compared to spontaneous pregnancies (p < 0.001). βHCG in the IVF/ICSI groups was significantly higher than the control group (p < 0.005). Center-specific modifications may be required to adjust screening parameters for ART.
**Bonne ** ***et al.*** **, 2016 (28)**	France	Retrospective cohort	Investigating the effect of assisted reproductive techniques on serum markers of Down syndrome in the first trimester of pregnancy.	Group A included 6621 patients with regular menses, whose menstrual cycles were calculated based on the date of the LMP. Group B included 529 patients whose pregnancy was achieved by IVF.	There was a significant difference in biochemical markers for trisomy 21 as a detriment to group B with a significant decrease in PAPP-A. CRL appeared to be longer, PAPP-A MoM decreased and hCG MoM increased. Specific ultrasound curves for pregnancies conceived with ART will be more relevant and accurate.
**Sahraravand ** ***et al.*** **, 2016 (29)**	Finland	Retrospective cohort	Evaluation of serum levels of ADAM12-s, PAPP-A and fβhCG in ART pregnancies.	283 ART pregnancies and 1008 spontaneous pregnancies.	ADAM12-s levels increased in all types of ART groups, while PAPP-A levels decreased only in the ICSI group compared to the control group. fβhCG levels did not differ between the 2 groups. Adjustment of screening parameters for ART should be considered.
**Savasi ** ***et al.*** **, 2015 (30)**	Italy	Prospective cohort	To investigate maternal serum levels of free βhCG and PAPP-A in the first trimester between OD pregnancies, IVF/ICSI with autologous oocytes, and natural pregnancies.	13,624 natural pregnancies, 171 pregnancies resulting from egg donation (IVF/ICSI), and 76 pregnancies resulting from IVF with autologous eggs (autologous IVF/ICSI).	Free βhCG levels in both OD pregnancies and autologous IVF/ICSI was significantly higher (p < 0.05) compared to the control and age-matched groups. PAPP-A levels were not significantly different among the 4 groups. NT was significantly lower in controls compared to OD IVF/ICSI (p < 0.05). These changes may be due to the IVF method.
All assessment tools were based on the STROBE Checklist except for references (37, 21, 32) which are based on the CONSORT Checklist. ART: Assisted reproductive technologies, PAPP-A: Pregnancy-associated plasma protein, CVS: Chorionic villus sampling, IVF: In vitro fertilization, ICSI: Intra cytoplasmic sperm injection, IUI: Intra uterine insemination, AFP: Alpha-feto protein, hCG: Human chorionic gonadotropin, βhCG: β-human chorionic gonadotropin, fβhCG: Free β-human chorionic gonadotropin, uE3: Unconjugated estriol, cfDNA: Cell free DNA, cffDNA: Cell-free fetal DNA, DS: Down syndrome, MSS: Maternal serum screening, PPV: Positive predictive value, NT: Nuchal translucency, FPR: False positive rate, FET: Frozen embryo transfer, CRL: Crown-rump length, NIPS: Non-invasive prenatal screening, LMP: Last menstrual period, ADAM12-s: A disintegrin and metalloprotease domain 12					

## 4. Discussion

The present study systematically reviewed all types of prenatal imaging and serum screenings for the diagnosis of Down syndrome in ART pregnancies to know and correctly interpret the results of prenatal screenings in these pregnancies. In the quality assessment of the descriptive studies included in this review, all the studies were of excellent quality and their score was higher than 16; the studies with moderate and poor quality were excluded from this systematic review. Nonetheless, review papers with similar titles did not pay attention to the quality of the included papers (46, 47).

Based on the results of the studies, prenatal screenings for Down syndrome in ART pregnancies can be divided into 2 categories: “first round screening" and “second round screening".

In the first-round screening after most types of ART methods, PAPP-A was significantly lower than spontaneous pregnancies, while free βhCG was significantly higher, especially in IVF and ICSI methods. Some studies also indicated an increase in NT in ART pregnancies. Regarding the biochemical markers of the second-round screening of Down syndrome, an increase in inhibin A and a decrease in AFP and UE3 are evident in some studies. These markers, which cause the Down syndrome screening test to be positive, can be present in ART pregnancies due to the presence of exogenous ovulation-stimulating hormones, the presence of multiple corpora lutea, twins or multiplets, the type of IVF method, and changes in the egg cytoplasm in the ICSI method.

This imposes unnecessary adjunct invasive procedures on the mother. Some studies have suggested screening through NIPT, cell-free DNA, or an integrated screening test to solve this false positive. Some others have also recommended the modification and adjustment of serum markers in Down syndrome screening for these mothers. The American College of Medical Genetics and Genomics has recommended the NIPT method for the diagnosis of trisomy 21, 18, and 13 as an alternative to traditional biochemical screening tests (48).

Nevertheless, there are false positives in these 2 methods. The American College of Obstetricians and Gynecologists and the Gynecologists' Committee on Genetics (2012) acknowledge that since cell-free DNA originates from the placenta and in sporadic cases as when the mass of the placenta is larger for some reason after ART, its values may be reported as falsely higher (43).

Among significant issues in the interpretation of screening tests for chromosomal disorders is that patients who refer for ART are usually older than those who conceive spontaneously. Hence, it is more likely that the mother carries a fetus with chromosomal disorders (49, 50). Another point is that among the types of ART methods, the ICSI method has a higher risk of transmitting chromosomal abnormalities; this issue emphasizes the importance of genetic evaluation of men before performing this method, because paternal characteristics may be the main reason for these chromosomal abnormalities (42).

These complications, along with reporting false-positive results, can raise the level of stress and anxiety in patients and doctors. Yet, in many cases, everything is normal and due to placental and corpus luteum disorders or differences in the type of method used; the results have been falsely positive. Some studies suggest that high fβHCG and low PAPP-A in cases where there are pregnancy complications, such as pre-eclampsia and fetal growth restriction can evidently be a sign of an abnormal placenta and explain the reason for the false positive of the Down syndrome test (26, 30, 51, 52).

Adjustment of biochemical markers for conception type is already included in the aneuploidy risk assessment algorithm by FMF (www.fetalmedicine.org); however, to optimize the performance of the screening test, the concentration of free βhCG and PAPP-A should be adjusted differently for each ART method. Regarding IVF and ICSI, such settings may especially limit false positives due to increased free βhCG and decreased PAPP-A. This may diminish the use of additional invasive methods that may impose additional costs on the couple and even lead to the risk of miscarriage and abortion (4, 18, 21, 23–25, 27, 29, 35, 37, 38, 53).

Smith-Bindman et al. by reviewing the meta-analysis of 56 articles and 1930 fetuses regarding prenatal ultrasound examination of Down syndrome in fetuses in the population of pregnant women, they concluded that although NT examination can be effective in prenatal diagnosis of Down syndrome fetuses, the overall sensitivity of this finding for it is too low to be a practical screening test in cases where the fetus has no associated structural abnormalities for Down syndrome. The use of these markers as a basis for the decision to perform amniocentesis leads to more fetal losses than in cases of Down syndrome and leads to a decrease in the prenatal diagnosis of fetuses with Down syndrome (54). This study is similar to the present study in terms of prenatal sonographic markers of Down syndrome in fetuses, but our study considered both serum and imaging markers and limited it to the population of women whose pregnancy was the result of ART.

Cavoretto et al., conducted a systematic review and meta-analysis on Down syndrome diagnosis markers including NT, free βhCG, and PAPP-A in IVF/ICSI pregnancies and reported similar results to the present study, that is, differences among the serum markers of prenatal diagnosis in ART pregnancies. They reported that free βhCG values were slightly higher in the ICSI group and PAPP-A values were slightly lower in the ICSI group, and also stated that these results may be due to changes in the placenta in ART pregnancies (55). The difference between the present study and this study is that we included a wider range of serum and imaging markers in both screening sessions in all types of ART methods in the study and we did a systematic review of the results of all of them and the need for different settings. We proposed the values of the markers mentioned in prenatal screenings for Down syndrome in pregnancies resulting from ART to reduce false-positive results and avoid the need for invasive diagnostic procedures. But due to the heterogeneity of the studies, it was only possible for the researchers to conduct a systematic review.

### Strengths and limitations

One of the strengths of the present systematic review was that it was the first systematic review that evaluated both serum and sonographic markers in both screening rounds for prenatal diagnosis of Down syndrome in post-ART pregnancies. However, due to the limitation of the data of the studies included in the present review, it was not possible to conduct a meta-analysis.

It is suggested to carry out future research in the form of a meta-analysis review to investigate the definitive effect of each type of assisted reproduction method on serum and sonographic markers in the prenatal diagnosis of Down syndrome.

The knowledge translation of this study can be prepared in the form of an educational materials (educational pamphlet, video, and like these) for the awareness of service providers to enhance cautious interpretation and the method of transferring information about the results of the first and second trimester screenings for prenatal diagnosis of Down syndrome in ART pregnancies to couples as well as ART service customers.

## 5. Conclusion

This systematic review found differences in the level of biochemical markers of Down syndrome screening and NT between ART pregnancies and spontaneous pregnancies, which can be due to various reasons such as placental disorders, corpus luteum multiplicity, and the type of ART method. Ultimately, these factors may cause false positive results, imposing aggressive procedures on these women for accurate diagnosis. Consequently, to optimize the performance of the screening test, the concentration of maternal serum markers such as free βhCG and PAPP-A, should be set differently for each ART method, especially between IVF and ICSI. However, more research is needed for strategies to reduce false positives in ART pregnancies.

##  Data Availability

Not applicable.

##  Author Contributions

FZ. Meamar: Searching in databases, writing proposals, writing the manuscript. M. Savabi-Esfahani: Searching in databases, writing the manuscript. T. Farajkhoda: Searching in databases, writing the proposal, and writing the manuscript.

##  Conflict of Interest

The authors declare that there is no conflict of interest.
